# Adherence to Iron-Folic Acid Supplementation and Associated Factors among Pregnant Women in Kasulu Communities in North-Western Tanzania

**DOI:** 10.1155/2020/3127245

**Published:** 2020-06-04

**Authors:** Winfrida B. Lyoba, Joyce D. Mwakatoga, Charles Festo, Jackline Mrema, Ester Elisaria

**Affiliations:** ^1^Department of Impact Evaluation, Health System and Policy Analysis, Ifakara Health Institute, Dar es Salaam, Tanzania; ^2^Department of Global Health and Bio-Medical Sciences, College of Life Science and Bioengineering, The Nelson Mandela Institution of Science and Technology, Arusha, Tanzania; ^3^Department of Agricultural Extension and Community Development, College of Agriculture, Sokoine University of Agriculture, Morogoro, Tanzania

## Abstract

**Introduction:**

Pregnant women are at a high risk of anaemia, with iron-folate deficiency being the most common cause of anaemia among pregnant women. Despite the well-known importance of iron and folic acid supplementation (IFAS) during pregnancy, adherence to these supplements is relatively low and associated factors were not well identified in the study area. This study is aimed at investigating adherence to IFAS and associated factors among pregnant women in Kasulu district, north-western Tanzania.

**Methods:**

A health facility cross-sectional survey with a mixed-method approach was conducted in Kasulu district from March to April 2019. A structured questionnaire was given to 320 women with children aged 0-6 months to assess factors associated with adherence to IFAS among pregnant women. Data were entered into SPSS version 22.0 for analysis. Binary logistic regression was further employed to determine the factors associated with adherence to IFAS. Focus group discussions were done with 19 pregnant women and 15 mothers of children aged 0-6 months to obtain more clarifications on the factors associated with adherence to IFAS. Furthermore, in-depth interviews were done with six health care providers to explore their perceptions of IFAS.

**Results:**

Out of the 320 respondents of the survey, 20.3% (*n* = 65) adhered to IFAS. Factors associated with adherence to IFAS among pregnant women included time to start ANC (AOR = 3.72, 95% CI: 1.42, 9.79), knowledge of anaemia (AOR = 3.84, 95% CI: 1.335, 10.66), counseling on the importance of the iron-folic acid (AOR = 3.86, 95% CI: 1.42, 10.50), IFAS given during clinical visit (AOR = 15.72, 95% CI: 5.34, 46.31), number of meals consumed (AOR = 3.44, 95% CI: 1.28, 9.21), number of children (AOR = 3.462, 95% CI: 1.035, 11.58), and distance to health facility (AOR = 0.34, 95% CI: 0.131, 0.886). Qualitative findings revealed that delayed first ANC visit, lack of remainder for pregnant women to take IFAS, low awareness about the negative effects of anaemia, low of knowledge of IFAS and management of side effects, negative beliefs about the use of IFAS, and follow-up mechanism were major reasons for poor adherence.

**Conclusion:**

Adherence to iron-folic acid supplementation during pregnancy was low. Strengthening systems for creating reminding mechanism, raising community awareness through educational programs to pregnant women and health providers could improve adherence to IFAS.

## 1. Introduction

Anaemia affects more than two billion people, 30-50% of whom are from developing countries including Tanzania [1]. Nearly 50% of all anaemia can be attributed to iron deficiency [1, 2]. Folic acid is also a vital micronutrient required for the metabolism, foetal growth, and the development of the neural tube [3]. Globally, the estimated prevalence of anaemia among nonpregnant and pregnant women was 29% and 38%, respectively, whereas, in Africa, the estimated proportions were 37.8% and 46.3%, respectively [2]. In Tanzania, the burden of iron deficiency accounts for approximately 57% among pregnant mothers and 46% among breastfeeding mothers [4]. Similarly, the prevalence of anaemia among women of reproductive age between 15 and 49 years is 45% in Tanzania and Kigoma region alone disproportionately carrying over half (55.1%) of the burden [4].

Pregnant women are more vulnerable to anaemia because of various factors. These include biological changes (menstrual period), undernutrition attributed to poverty, food insecurity, gender inequalities, inadequate knowledge of proper dietary practices, and increased iron and folic acid deficiency [5]. Furthermore, pregnant women experience increased micronutrient demand especially iron for the growth of foetus and metabolism which cannot be easily met by diet alone because of the poor intake and low absorption of iron [3].

Women who failed to consume the tablets according to the IFAS recommendation would experience iron and folic acid (IFA) deficiency anaemia and associated negative effects on mothers and newborns. Folic acid deficiency in pregnant women is associated with preeclampsia and, preterm delivery. It is also related to neural tube defects, foetal malformation, type 2 diabetes, and obesity in the newborn [6–8]. Similarly, iron deficiency anaemia is connected with maternal risks including haemorrhage, premature delivery, rupture membrane, decreased work capacity, and maternal deaths. Correspondingly, small for gestational age, low birth weight, poor cognitive development, stillbirth, and cardiovascular disease were risks associated with iron anaemia in the newborn [2, 9, 10]. Anaemia leads to approximately 50,000 deaths during childbirth each year globally [9] and iron deficiency alone accounts for 22% of all anaemia deaths [7]. In sub-Saharan Africa, anaemia contributed to approximately 28.6% of maternal deaths [11] and 10% in Tanzania [12].

The World Health Organization (WHO) recommends uptake of iron and folic acid supplement during pregnancy for at least six months [13]. Tanzania adopted the WHO recommendation and requires all pregnant women to consume 30-60 mg of a dose of iron and 400 *μ*g of folic acid daily starting early at first antenatal visit [2, 4, 13]. Despite the national guideline emphasis, adherence to iron-folic acid supplements during pregnancy is still low [4].

The proportion of pregnant women who reported to uptake iron and folic acid supplementation in Tanzania for at least 90 days was less than a quarter (21%) with only 7% in Kigoma region [4]. IFAS is considered the best strategy to control anaemia during pregnancy, but low adherence to IFAS compromises the effectiveness of this strategy [4]. Based on these facts, describing adherence to IFAS and associated factors among pregnant women would be the potential for evidence-based interventions.

Moreover, various studies have been conducted in Tanzania about IFAS; however, the majority of these focused on the effects of IFAS in newborn and pregnant women and only limited studies assessed the adherence to IFAS and associated factors among pregnant women. With these inadequate studies, the available data may also be outdated. Currently, studies conducted on IFAS in Kasulu district are limited. Therefore, this study is aimed at assessing adherence to IFAS and associated factors in the Kasulu district in north-western Tanzania.

## 2. Methods and Materials

### 2.1. Study Design and Study Area

A health facility-based cross-sectional study using a mixed-method approach was conducted in Kasulu district in Kigoma region, 4°44′0° S and 30°44′0° E Tanzania from March to April 2019 [14]. The district has two administrative areas with a total population of 634,038 people [15]. Kasulu district is predominately occupied by the “Waha” ethnic group. The main activities are subsistence and small-scale farming and medium-scale business, particularly in Kasulu town. The other tribe found in the district is Sukuma, who engaged in pastoralism activities [14]. The average of pregnant women registered in Kasulu district per year was 38,951, and nearly three quarters were from Kasulu rural. A total of 34,365 of women with children aged 0-6 months were also recorded, and over seventy percent were from Kasulu rural.

### 2.2. Study Population

**Figure 1:**
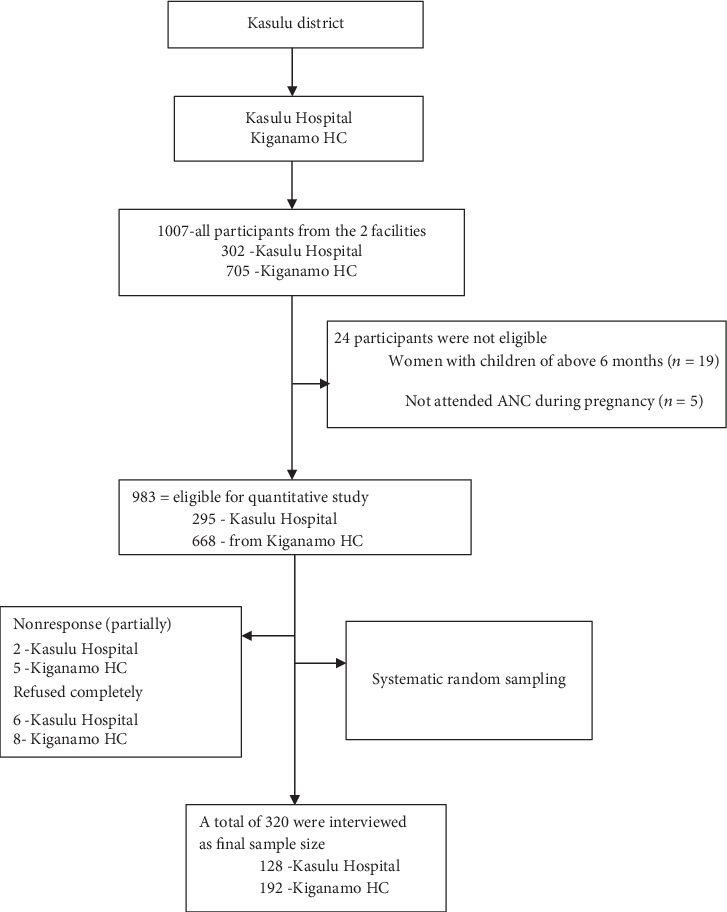
Participants' flow diagram.

The study population consisted of health care providers, pregnant women, and mothers with children aged 0-6 months, who attended antenatal clinic (ANC) and postnatal services during the study. Women with children aged 0-6 months, attended ANC visits during their last pregnancy, and supplied with IFAS were included in the quantitative study. The qualitative study involved pregnant women, women with children aged 0-6 months, and health care providers working at the reproductive and child health department. Health care providers and all mothers came from two health facilities in Kasulu district; these were Kasulu Hospital (KH) and Kiganamo Health Center (KHC). All women with children aged 0-6 months who did not attend ANC visit during pregnancy, all women having less than three ANC visits, and all women who were not supplied with IFAS, unable to speak/hear, or with mental disorders were excluded from the study. Detailed information in Figure 1.

### 2.3. Sample Size and Sampling Procedures

A total of 320 mothers of children aged 0-6 months were included in the study. The sample size was determined based on a single population proportion formula (*Z*^2^*P*(1 − *P*)/(*e*)^2^) with a 95% Cl, 5% margin error (*Zα*/2 = 1.96) by considering the national adherence to IFAS (*P* = 21%) [4] at 80% desired power (*Bα*) and 10% of the estimated nonresponse rate. Nonresponse was considered to maximize the sample size of 320 study respondents.

Mothers of children aged 0-6 months were systematically sampled from a list of clients attending postnatal services in the two facilities. These included Kasulu Hospital and Kiganamo Health Center which are well known to accommodate and provide maternal services to a large number of pregnant women compared to other health facilities in Kasulu town. This study was done between March and April 2019. In addition, 40 respondents were purposively selected and involved in a qualitative study. These included six health care providers who participated in in-depth interviews (IDIs), 19 pregnant women, and 15 mothers with children aged 0-6 months who participated in focus group discussions (FGDs). A total of four FGDs were conducted of which two comprised of pregnant women and two comprised of mothers of children aged 0-6 months. Each FDG included 7-11 respondents.

### 2.4. Data Collection Procedures and Quality Control

A structured questionnaire established based on information from Tanzania Demographic Health Survey (TDHS) and published articles was administered to women who had children aged 0-6 months [4, 16–18]. The structured questionnaire captured sociodemographic information, maternal factors, health facility factors, household factors, medication factors, access to health care, perception of the quality of services, knowledge of anaemia, knowledge of IFAS, and adherence to IFAS. All tools were developed in English and translated into Swahili (a local language) for simplicity and understanding and then back-translated to English by language researchers conversant in both languages to facilitate consistency. Data collectors and supervisors were trained on the objectives, how to keep confidential information, and filling of information. Tools were pretested, and all necessary changes were made before the start of actual data collection. An interview guide was used for the focused group discussion and in-depth interviews.

### 2.5. Operational Definition

#### 2.5.1. Adherence to IFAS

Adherence to IFAS was self-reported and assessed by considering the total numbers of IFAS tablets consumed during their last pregnancy. Pregnant women were recommended to consume one tablet of iron and folic acid tablets containing 30-60 mg of iron and 400 *μ*g of folic acid daily for at least 90 days [2, 4, 13]. Six questions were added together to assess adherence to IFAS. These include information about the number of ANC visits, the number of sachets given in each visit, and the total number of tablets given for the whole period of pregnancy. The number of tablets consumed and remaining before childbirth was also recorded. All women who took tablets for at least 90 days were considered to adhere to the IFAS program and vice versa.

#### 2.5.2. Knowledge of Anaemia

The knowledge of anaemia was assessed by adding up the four multiple-choice items including causes, symptoms, consequences, and prevention methods of anaemia. The correct answer was labeled, and the wrong answer was not labeled. Answers were categorized into two groups; those who scored medium and above were considered to have high knowledge and low knowledge when scored below medium.

#### 2.5.3. Knowledge of Iron-Folic Acid Supplementation

To understand the knowledge of IFAS among respondents, six multiple-choice questions were added together including physical appearance (color), time to start using IFAS, the recommended number of tablets, benefits of IFAS, and negative effects to mothers and children when the adequate tablets were not consumed. The correct answer was labeled, and the wrong answer was not labeled. Answers were classified into two groups and labeled high knowledge if scored medium and above and low knowledge of IFAS if scored below medium.

#### 2.5.4. Perception of the Quality of Services

To measure perception, five multiple-choice questions were added up including the number of tablets provided, general physical checkup, service satisfaction, being given IFAS for free, and customer care. The answer was ordered into two groups and labeled positive perception when scoring medium and above and negative perception when scoring below medium.

### 2.6. Data Processing and Analysis

Quantitative data were checked, coded, and entered into SPSS software version 22 with a statistical significance decided at *P* value < 0.05. Descriptive analysis, including frequencies, percentages, and chi-square testing, was employed when determining the association among variables and adequacy of cells. A binary logistic regression model was fitted after multicollinearity diagnosis to establish factors to IFAS adherence. Adjusted odds ratio (AOR) with 95% CI, *P* value < 0.05 was considered statistically significant.

The recall bias was managed through interviewing the mothers who gave birth within a short time, six months prior to the study (women with children aged 0-6 months). Also, a sachet of iron and folic acid supplementation was demonstrated to mothers during the interview to aid their memories.

All audio records from qualitative interviews were transcribed and translated to English before analysis. The authors transcribed each discussion verbatim and carefully checked each transcript for accuracy by simultaneously listening to the audio recording and reading transcript. Notes taken during the discussions or interviews were incorporated in the final transcripts. Data were coded and analyzed using NVIvo software version 10. Thematic analysis was employed to explore the themes raised during focus group discussions and in-depth interviews. Finally, the themes that stemmed from both FGD and IDI were triangulated with quantitative findings.

### 2.7. Ethical Consideration

Ethical clearance and approval of the study were obtained from Ifakara Health Institute Review Board on 9^th^ February 2019 (IHI/IRB/No: 9-2019). Introduction letter from the Department of Research and Training of Ifakara Health Institute was also provided to investigators. The study team made a courtesy visit to the district administration prior to data collection. Furthermore, the purpose of the study was explained to all respondents and written informed consent forms were provided and signed before the interview. The verbal consent was also enquired before the group discussion. The informed consent was read to respondents loudly in front of the witness. Respondents who were unable to read and write were asked to sign using a thumb fingerprint before the study. The confidentiality of their information was assured among respondents.

## 3. Results

### 3.1. Social and Demographic Characteristics of Women with Children Aged 0-6 Months

The summary of the sociodemographic information of the respondents is provided in Table 1. Of the 320 respondents of the survey, 128 were from Kiganamo Health Center and 192 from Kasulu Hospital. A majority of the respondents were aged 15-24 years 140 (43%). More than half of the respondents (53.75%) completed primary education, and nearly three quarters (*n* = 232, 72.5%) were not employed in formal sectors (Table 1).

### 3.2. Knowledge about Anaemia

Out of 320 respondents, two hundred twenty-two (69.4%) had high knowledge of anaemia. The findings from qualitative study concerning the causes of anaemia were thought to be sitting under the sun, infectious diseases (malaria, UTI, HIV, and worms), poor diet, and inadequate consumption of vegetables and fruits. The symptoms of anaemia include dizziness, fatigue, feeling sick, and edema. The majority of respondents found that the consequences of anaemia among pregnant women were death attributed to bleeding during childbirth and miscarriage. On the other hand, health care providers had different understanding regarding the meaning of anaemia in pregnant women. Anaemia in pregnant was implied when Hb was below 7.5 mg/l, and others said pregnant women were considered to have anaemia when Hb was below 12 mg/l.

### 3.3. Knowledge of Iron-Folic Acid Supplementation (IFAS)

Awareness of IFAS among women with children aged 0-6 months was demonstrated by attending antenatal clinics. Among all respondents, less than half (*n* = 110, 34.4%) had high knowledge of IFAS and were primarily received during the ANC visit. A total of two hundred fifty-nine (80.9%) women had an understanding that IFAS increases blood level during pregnancy. Similarly, seventeen women (5.3%) had knowledge of IFAS that it works best to improve the health of the newborn. Qualitative findings revealed that a belief on IFAS to increase blood level influenced the women's adherence to IFAS. It was also reported that deaths and abnormalities like neural tube defects among newborns were reduced when IFAS is consumed properly during pregnancy. On the other hand, pregnant women built trust in the performance of health care providers. In the same manner, both women and health care providers had low awareness about the timing of starting IFAS. Further, the time spent to educate women about the importance of IFAS was not enough (30 minutes) as quoted below:

“We could not provide a detailed explanation to pregnant women; we often tell them, IFAS prevents you from giving birth to a child with a big head, but when asked how it works, it becomes difficult to explain because we have no further information”. …..She emphasized, “We need to update our knowledge” (a health care provider at KH).

### 3.4. Factors Related to Adherence to Iron-Folic Acid Supplementation among Pregnant Women

Over ninety percent of respondents (*n* = 296) were given IFAS from two clinics, and the adherence to IFAS was 20.3% (*n* = 65). The reasons related to poor adherence were delayed initiation of ANC, distance to the health facilities, availability of IFAS, perceived quality of health facility services, being healthy, women's experiences with multiple births, misconception about pregnancy and IFAS, lack of knowledge on the benefits of IFAS, negligence, forgetfulness, and side effects, including nausea, dizziness, and vomiting. See the quote below:

“If not sick, why should we take it”. “….we were also told by women in our communities that, whoever takes IFAS, experiences more bleeding during delivery and gave birth to blaze skinny babies” (FGD: women with a child aged 0-6 months at KHC).

#### 3.4.1. Timing of ANC Services

Most of the women, approximately two hundred and seventeen (67.8%), started the first antenatal clinic (ANC) visit during the second or third trimester (Table 2). Delayed ANC attendance was associated with women's perception. Attending to ANC early was attributed with the negative perception that woman was weak, fear of being pregnant, and pregnancy was designated as secret. It was also associated with notion around HIV testing to both partners as part of prevention of mother-to-child transmission (PMTCT) program accompanied by partners to attend ANC as quoted below:

“We want to be sure of what is inside the womb by sensing fetal movement first before going to the clinic for the first visit because if you go early many will laugh at you and say it's like you are making an advertisement that you have pregnant, it is a show up” (FGD: women with children aged 0-6 months at KH).

During the discussion, a prime woman with the age of 28 years emphasized that the PMTCT program and related attributes were associated with delayed ANC visits.

“The first time I came to the clinic, I found a case of pregnant women who came with a motorcycle driver acting to be her husband. After testing for HIV, a driver was infected. During the counseling session, a pregnant woman confirmed that a man was not her husband. Therefore, this strategy puts pressure on women leading them to tell lie and delay to attend ANC services” (FGD: pregnant women at KH).

#### 3.4.2. Distance to the Health Facility

Distance was estimated by using the time spent from residents to the health facility. Over half of the women spent 30 minutes or less to reach the health facility (*n* = 172, 53.8%), and one hundred forty-eight respondents spent 60 minutes and above (46.2%) as presented in Table 2. On the other hand, the cost of transportation is attributed between 1000 and 3000 Tshs per each ANC visit. The challenge of distance to attend ANC during pregnancy was also revealed from qualitative findings as quoted below:

“Please help us to have a clinic close to residential areas, it is difficult for a pregnant woman to walk more than one hour to access ANC services” (FGD: pregnant women at KHC).

#### 3.4.3. Support from a Partner

Nine percent (*n* = 28) of women reported being reminded by their husband about taking IFAS during pregnancy (Table 2). A similar finding was reported in the qualitative data as quoted below:

“How do they remind us when they do not understand its benefits…, they even not bother…, they don't care…, they are less concerned” (FGD: pregnant women at KH).

#### 3.4.4. Perception of the Health Services

Nearly three-quarters of the women (*n* = 230, 71.9%) had a positive perception of health services provided at health facilities (Table 2). A majority of the respondents perceived the quality of services provided at both health facilities to be good. Challenges reported were much time spent at the ANC that was attributed to the large number of women presented at each visit and an inadequate amount of tablets provided at each visit that sometimes was not enough to meet the next visit. It was also reported that being instructed to come with partners at ANC during the first visit was another challenge thought by respondents. These were different to health care providers; challenges of the IFAS program and services were attributed with a few staffs and poor working environment as quoted below:

“IFAS does not belong to anybody, because other services like malaria, family planning, and HIV have special people working closely with the ANC and community to ensure the community understands better. No effort has been put in place to advocate on the importance of IFAS in and out of clinics” (a health care provider at KH).

#### 3.4.5. Availability of IFAS

IFAS was provided at ANC for free; however, forty percent (*n* = 128) of the respondents attended ANC visits only were given IFAS on each visit. Women who were provided 90 IFAS tablets or more was low compared to the women who were given IFAS during their ANC visit (*n* = 296, 92.5%) with frequently IFAS stock out in health facilities.

### 3.5. Factors Associated with Adherence to Iron-Folic Acid Supplementation (IFAS) among Pregnant Women Attended ANC Services

In this study, IFAS given in each visit, knowledge of IFAS, knowledge of anaemia, gestation period at first ANC visit, the number of meals, and number of children were the factors associated with adherence of IFAS among pregnant women. The details on the associated factors of IFAS are shown in Table 3.

## 4. Discussion

In this study, the adherence to IFAS was low among pregnant women and factors associated with adherence to IFAS were included: the knowledge of anaemia, knowledge of IFAS, time a woman attended at ANC visit, number of IFAS tablets provided, number of meals, number of children, and distance to the health facility. Details are found in Table 2.

The result of adherence to IFAS in this study was consistent with national adherence (21%) [4]. Similarly, studies conducted in Kiambu, Kenya (32.7%), and Amhara, Western Ethiopia (20.4%), also found the low adherence to IFAS among pregnant women [19, 20]. Also, adherence to IFAS was modestly higher compared to the studies conducted in Eastern Kenya (18.3%), Uganda (12%), Norway (16%), and Northern Tanzania (16.1%) [21–24] and lower compared to studies conducted in Senegal (51%), Kathmandu-Nepal (73.2%), Tamil Nadu-India (60.6%), and Mizan-Aman-Ethiopia (70.6%) [25–28]. Some of the probable reasons of inconsistency might be a different study subject, sociodemographic characteristics, locality, follow-up and reminding mechanism, knowledge of IFAS, and qualities of health services offered in the health facilities including counseling on its benefits, the average number of IFAS recommended during pregnancy, and belief on the use of IFAS.

The findings of this study indicated that women who had high knowledge of IFAS were 3.9 times more likely to adhere to the IFAS program compared to the women who had low knowledge. The study was reliable with the studies conducted in Northern Wollo, Ethiopia, and Misha district, Ethiopia [29, 30]. The time spent to counsel pregnant mothers about the benefits of IFAS, consequences of not using IFAS, proper prescription, and awareness of side effect management were the reasons for adherence to the IFAS program.

Women with high knowledge of anaemia were 3.8 times more likely to adhere to the program compared to women with low knowledge of anaemia. This was consistent with studies conducted in Aykel, Ethiopia, and Nepal [26, 31]. The probable reasons for adherence might be the proper counseling about the prevention measures, consequences of anaemia at ANC, and availability of media used to create awareness in communities.

Similarly, women provided with IFAS tablets in each visit were 15.7 times more likely to adhere to the program compared to women who were not provided with IFAS each visit. These findings corroborated with studies conducted in Mizan-Aman, Ethiopia, and Pakistan [28, 32]. Studies conducted in Uganda, Pakistan, and South Africa found that less supply of IFAS had low adherence to IFAS [24, 32, 33]. Other studies conducted in Ismailia government and Assela town-Ethiopia reported that crowding of tablets was associated with the low adherence to supplementation [34, 35]. The reasons might be associated with the availability of tablets at ANC, the number of ANC visits, knowledge of health care providers about time to start IFAS, and awareness of the average number of IFAS required for adherence.

Other studies have indicated that women with high wealth quintile and eating three and above meals per day were likely to adhere to IFAS compare to women from low wealth quintile [36, 37]. However, in the present study, women from the families eating twice or less were found to be 3.4 times more likely to adhere to IFAS as compared to women from the families who ate three times and more. This was contrary to a study conducted in India, which showed that women from households with high wealth quintile were less likely to adhere to IFAS due to good health and able to access them when needed [27]. The reasons might be education level, occupation of women and their partners or household income, location, and verse versa.

The time to start ANC visits was associated with adherence to the IFAS program. The present study revealed that women who attended ANC visit in the first trimester were likely to adhere to the IFAS program 3.7 times more compared to those who started ANC visit in the second trimester and third trimester. This was consistent with studies conducted in North Wollo Zone-Ethiopia, Ibadan-Nigeria, Bangladesh, and Tamil Nadu-India, which reported that early registration in the first and second trimester has a positive effect to adherence to IFAS program than in the third trimester [16, 27, 29, 38]. The possible reasons for adherence might be the knowledge on the time to start ANC, beliefs, perception, distance to the health facility, and delayed ANC visit attributed to HIV testing to both women and their partners. This might affect directly the duration and number of tablets consumed to adhere to the IFAS program.

In this study, women with 1-3 children were 3.5 times more likely to adhere to the program compared to women with 4 and above children. This was consistent with studies conducted in India and Tanzania, which showed that women with two children and below were likely to adhere to the program compared to women with more children [21, 39]. According to a study conducted in Assela town, Ethiopia, it was reported that having fewer children had less likely to adhere compared with women with more children [35]. The probable reasons for adherence to IFAS could be the maternal-related experience. Studies conducted in Debre Tabor-Ethiopia and Ismailia government reported that gravidity had an association with adherence to IFAS [17, 34].

The distance from residential to the health facilities was associated with adherence to the IFAS program. Women living 60 minutes or more from the health facilities were less likely to adhere to the IFAS program 0.34 times compared to the women living near the health facilities. In other studies, women who were living close to health facilities or private pharmacies had more chance to adhere to the IFAS program more times compared to women who were living far from health facilities [25, 36, 40]. The reasons might be the lack of close health facilities and less economic index; less availability of pharmacies used to sell IFAS and negligence among mothers were the reasons for low adherence to the IFAS program.

The other reasons for low adherence to IFAS were revealed from the qualitative analysis including women's experiences, lack of knowledge about the benefits of IFAS, negligence, forgetfulness, and the side effects including nausea, dizziness, and vomiting. The other reason was misinformation about pregnancy and misperception around the IFAS, including giving birth to a child with burned skin. It was also reported that the majority did not consume IFAS if they were feeling healthy and become less motivated. This study corroborates with the study conducted in Ethiopia [41, 42]; however, the other studies reported that fear of being sick or being sick was associated with adherence to the IFAS program [21, 26, 28, 43]. On the other hand, misinformation included fear to give birth to big baby, spot-on teeth of children and face of mothers, difficult delivery, not beneficial to babies, mothers experience with no history of having children with neural tube defects, and more bleeding during delivery was associated with low adherence to IFAS [10, 20, 33, 35, 44]. Regarding forgetfulness, the study was consistent with various studies conducted in various regions including Kenya, Vietnam, Pakistan, Ethiopia, and Iran [23, 32, 43, 45, 46].

### 4.1. Limitation of Study

The study had its limitations and needs to be considered during the interpretation of the results. First, adherence to IFAS was determined by self-report which might affect the actual adherence due to the possibility of over- or underreporting of ingested tablets. Second, pill counts and measuring of haemoglobin concentration and biomarkers may predict better the adherence to IFAS; however, it was not done in this study.

## 5. Conclusion

Adherence to iron and folic acid among pregnant women was low. Factors associated with adherence to IFAS included time to start ANC, knowledge of anaemia among women, counseling on the importance of the iron-folic acid, number of IFAS provided each visit, and number of children. However, the side effects and distance to the health facility had less effect on adherence to IFAS. Therefore, the prevention of iron-folic acid deficiency through strengthening the system to create community awareness; health promotion and counseling; education programs among health care providers, males, and pregnant women; and reminding mechanisms is paramount to improve adherence to IFAS.

## Figures and Tables

**Table 1:** The sociodemographic characteristics of respondents in Kasulu district, north-western Tanzania, March-April 2019 (*N* = 320).

Variables	Categories	Frequency (*n*)	Percent (%)
Mothers age (years)	15-24 years	140	43.8
	25-34 years	121	37.8
	35-49 years	59	18.4

Mothers education	No formal education	69	21.5
	Completed primary	172	53.8
	Completed secondary +	79	24.7

Mothers occupation	Not employed	232	72.5
	Employed	88	27.5

Marital status	Single	35	10.9
	Married	285	89.1

Family size	1-3	89	27.8
	4-6	136	42.5
	6+	95	29.7

+ = Above.

**Table 2:** Characteristics related to supplementation among respondents in Kasulu district, north-western Tanzania, March-April 2019 (*N* = 320).

Variables	Categories	Frequency (*n*)	Percent (%)
Distance to the health facility (minutes)	0-30 minutes	172	53.8
	60+ minutes	148	46.2

Number of children	1-3 children	221	69.1
	4+ children	99	30.9

Time at first ANC visit	0-3 months	103	32.2
	4-6 months	197	61.6
	7-9 months	20	6.2

Access to IFAS	ANC	296	92.5
	Not ANC	24	7.5

IFAS given each visit	Yes	128	40
	No	192	60

Reminded by a husband to take IFAS	Reminded	28	8.8
	Not reminded	292	91.3

Side effects	Side effects	108	33.8
	No side effects	212	66.2

Knowledge of anaemia	High knowledge	222	69.4
	Low knowledge	98	30.6

Knowledge of IFAs	High knowledge	110	34.4
	Low knowledge	210	65.6

Time spent to educated IFAS	Enough	92	28.8
	Not enough	228	71.2

Perception of health services	Positive	230	71.9
	Negative	90	28.1

Adherence level	Adherence	65	20.3
	No adherence	255	79.7

**Table 3:** Factors associated with adherence of IFAS among respondents in Kasulu district, north-western Tanzania, March-April 2019 (*N* = 320).

Variables	Adherence level		COR (CI at 95%)	AOR (CI at 95%)
	Adhered *N* (%)	Not adhered *N* (%)		
Distance to the health facility (minutes)				
60+ minutes	27 (16.77)	134 (83.23)	1.271 (0.732, 2.205)	0.338 (0.131, 0.886)^∗^
0-30 minutes	38 (23.90)	121 (76.1)		1
Number of ANC visits				
4+	49 (24.62)	150 (75.38)	0.466 (0.256, 0.865)	0.6106 (0.220, 1.692)
Less than 4	16 (13.22)	105 (86.78)		1
Knowledge of IFAS				
High knowledge	35 (31.82)	75 (68.18)	2.80 (1.604, 4.890)	3.864 (1.422, 10.500)^∗^
Low knowledge	30 (14.29)	180 (85.71)		1
Knowledge of Anaemia				
High knowledge	56 (38.62)	89 (61.38)	3.336 (1.577, 7.059)	3.840 (1.335, 10.685)^∗^
Low knowledge	09 (5.14)	166 (94.86)		1
IFAS given each visit				
Given each visits	52 (40.62)	76 (59.38)	9.421 (4.848, 18.307)	15.718 (5.335, 46.311)^∗^
Not given each visits	13 (6.77)	179 (93.23)		1
Number of meals				
Twice and below	49 (28.32)	124 (71.68)	3.385 (1.721, 5.894)	3.439 (1.284, 9.212)^∗^
Three times or more	16 (10.88)	131 (89.12)		1
Time at first visit				
1^st^ trimester	33 (32.04)	70 (67.96)	2.725 (1.559, 4.765)	3.724 (1.417, 9.791)^∗^
2^nd^+ trimester	32 (14.75)	185 (85.25)		1
Side effect				
Side effects	12 (11.11)	96 (88.89)	0.375 (0.191, 0.757)	0.132 (0.043, 0.406)
No side effects	53 (25)	159 (75)		1
Reminded by husband				
Yes	55 (18.84)	237 (81.16)	2.394 (1.047, 5.473)	1.157 (0.454, 2.946)
No	10 (35.71)	18 (64.29)		1
Number of children				
Less than 4 children	57 (25.79)	164 (74.21)	3.185 (1.807, 8.651)	3.462 (1.035,11.582)^∗^
4+ children	8 (8.08)	91 (91.92)		1

AOR = adjusted odds ratio; CI = confidence interval; COR = crude odds ratio; ^∗^statistically significant; 1 = reference. Note Hosmer and Lemeshow Test = 0.925; therefore, the model is fitted to predict the factors associated with adherence.

## Data Availability

The data used to support the findings of this study are available from the corresponding author upon request.

## Abbreviations

ANC: Antenatal care
DHS: Demographic and Health Survey
DST: Daylight saving time
FGD: Focused group discussion
GMT: Greenwich Mean Time
HIV: Human infection virus
HSSP: Health Sector Strategic Plan
IDI: In-depth interview
IERB: Institutional Ethical Review Board
IFAS: Iron and folic acid supplementation
KH: Kasulu Hospital
KHC: Kiganamo Health Center
MSD: Medical Store Department
NBS: National Bureau of Statistics
PMTCT: Prevention of mother-to-child transmission of HIV
OR: Odds ratio
TDHS: Tanzania Demographic and Health Survey
URT: United Republic of Tanzania
UTI: Urinary tract infection
VIF: Variable interaction factor
WFP: World Food Program
WHO: World Health Organization.
